# The Consequences of Financial Leverage: Certified B Corporations’ Advantages Compared to Common Commercial Firms

**DOI:** 10.1007/s10551-023-05349-5

**Published:** 2023-02-14

**Authors:** Ine Paeleman, Nadja Guenster, Tom Vanacker, Ana Cristina O. Siqueira

**Affiliations:** 1 Faculty of Business and Economics, University of Antwerp, Prinsstraat 13, 2000 Antwerp, Belgium; 2 School of Business and Economics, Finance Center Muenster, University of Muenster, Universitaetsstr. 14-16, 48149 Muenster, Germany; 3 Faculty of Economics and Business Administration, Ghent University, Sint-Pietersplein 7, 9000 Ghent, Belgium; 4 Cotsakos College of Business, William Paterson University, 1600 Valley Road, Wayne, NJ 07470 USA; 5 University of Exeter Business School, Rennes Drive, Exeter, EX4 4PU UK

**Keywords:** B Corp social firms, Entrepreneurial finance, Social entrepreneurship

## Abstract

Firms usually need to attract debt to form and grow, but increasing financial leverage also entails increased risks and costs for stakeholders, such as customers and employees. Accordingly, past research suggests that for common commercial firms (CCFs), which prioritize profits, higher leverage leads to lower sales growth and higher employment costs. However, Certified B Corporations (CBCs) distinguish themselves by having a credible prosocial mission and, therefore, might be better insulated against the adverse effects of higher leverage. Using a European multi-country matched sample of 136 CBCs and 136 CCFs, we find that the negative relationship between leverage and sales growth and the positive relationship between leverage and employment costs are weaker for CBCs than CCFs. Taken together, due to their certified prosocial mission, CBCs enjoy an advantage in debt financing compared to CCFs.

**Supplementary Information:**

The online version contains supplementary material available at 10.1007/s10551-023-05349-5.

## Introduction

Certified B Corporations (CBCs) represent a growing global movement that uses traditional business and market-oriented models to address grand societal challenges, such as building more inclusive and sustainable economies (e.g., Branzei et al., 2018; Parker et al., 2019; Patel & Chan, 2021; Pollack et al., 2021; Villela et al., 2021). CBCs are hybrid companies: while they (need to) pursue profits, just like common commercial firms (CCFs), they also participate in social and environmental audits that are conducted by the independent, nonprofit B Lab (Moroz et al., 2018). This certification allows firms to formally commit to social and environmental goals beyond financial goals. As Moroz et al., (2018, p. 119) state: “CBCs are a distinct and readily identifiable set of businesses that epitomize the core aspects of social hybrid organizations… CBCs must publicly consider people, planet, and profit….”.^1^

CBCs are a specific set of social hybrid firms (e.g., Branzei et al., 2018; Gehman & Grimes, 2017; Grimes et al., 2018; Moroz et al., 2018; Sharma et al., 2018). Scholars have started to investigate the financing of social hybrid firms more broadly (Bruton et al., 2015; Calic & Mosakowski, 2016; Desa & Basu, 2013; Markman et al., 2016; Siqueira et al., 2018). Financing is important because it can critically affect firms’ scalability (OECD, 2018) and, therefore, their ability to address grand societal challenges. However, to date, we lack insights into the consequences of social hybrid firms’ financial structures; an issue we address in the current paper. This gap is problematic because the current theoretical perspectives on the consequences of firms’ financial structures, which mainly come from finance, depict people as primarily self-interested and focused on personal financial value maximization (Donaldson & Davis, 1991). However, we cannot simply generalize the insights from such perspectives to CBCs and social hybrid firms more broadly.

Indeed, CBCs’ utility functions are different than their common “commercial” counterparts. Certainly, CBCs also care about profits. As one entrepreneur in Conger et al., (2018, p. 189) states: “If we did everything for the greater good—we’d have to close the doors here”. However, CBCs are a distinct set of firms that usually have a strong prosocial mission (Conger et al., 2018). These firms’ stakeholders, such as customers and employees, often share and value CBCs’ mission (e.g., Austin et al., 2006; Bianchi et al., 2020; Conger et al., 2018; Winkler et al., 2019). Conversely, CCFs, which focus on profit maximization as a primary goal, can also have (secondary) social and environmental goals (e.g., Estrin et al., 2013). But while many firms talk about “doing good”, talk is cheap and does not necessarily represent the facts (e.g., greenwashing) (Parguel et al., 2011). Before getting certified, however, CBCs are carefully audited and the certification entails costs (Parker et al., 2019). Hence, CBCs’ certification could serve as an easily identifiable, credible signal of their prosocial mission toward stakeholders (Chauhan & O’Neill, 2020).

Accordingly, managers in CBCs and stakeholders can react differently to financial structures than their counterparts in CCFs. Therefore, we study the effects of leverage as a key aspect of a firm’s financial structure. We define leverage as the proportion of assets financed by debt with a maturity of more than one year. Previous research on CCFs, in which people are assumed to be primarily focused on maximizing financial wealth, emphasizes the negative consequences of leverage for customers and employees (Hortaçsu et al., 2013; Maksimovic & Titman, 1991; Moussu & Ohana, 2016). Accordingly, in CCFs, higher leverage can lead to losses in sales growth and increasing employment costs (Akyol & Verwijmeren, 2013; Titman, 1984). In this paper, the primary focus is on CBCs, which have a strong prosocial mission and stakeholders that share their non-financial goals, and we ask: *How does leverage relate to the sales growth and employment costs of CBCs in comparison to CCFs?*

Extant perspectives from finance highlight that self-interested entrepreneurs who prioritize personal wealth have incentives to transfer some of the costs of higher leverage to their customers and employees (Titman, 1984). In more highly leveraged firms, there is a higher risk of financial distress. Higher leverage and financial distress could affect (prospective) customers who might expect to lose warranties and product support, and it could even become financially optimal for entrepreneurs to decrease the safety and quality of their products and services (Kini et al., 2017; Maksimovic & Titman, 1991). Accordingly, self-interested customers could decide to buy from other firms and thereby hampers sales growth. Moreover, higher leverage also affects employees who face higher unemployment risk, earnings losses, and higher losses of firm-specific human capital. Therefore, self-interested employees, who optimize their own financial situation, could require higher compensation for bearing these risks (Berk et al., 2010). The combination of these “stylized facts” from the finance literature, which has often focused on US public firms, shows that leverage is negatively related to sales growth and positively related to employment costs.

We hypothesize that the above effects of leverage will be more likely to occur in CCFs but are less pertinent to CBCs and their stakeholders. We argue that the certification of CBCs ensures that these firms have a genuine goal to create a positive social impact (Cao et al., 2017; Doherty et al., 2014). Accordingly, CBCs may want to minimize the possible negative effects of higher leverage on employees and customers. Moreover, CBCs’ certification may serve as a visible and credible signal of their prosocial motivations (Cao et al., 2017; Parker et al., 2019) for (prospective) customers and employees. Accordingly, CBCs’ employees and customers—who frequently share the CBCs’ social mission (Austin et al., 2006)—may also be less inclined to require higher wages which decreases firms’ employment costs, or switch to other suppliers when the leverage and thus risk of the firm increases. Consequently, we hypothesize that the negative relationship between leverage and sales growth and the positive relationship between leverage and employment costs will be significantly weaker in CBCs than in CCFs.

To test our hypotheses, we constructed a unique database of 136 CBCs and 136 matched CCFs from six European countries (Italy, Spain, France, United Kingdom, Portugal, and Germany). We matched the CBCs with CCFs in the year of certification based on four characteristics: country, industry, age, and size (e.g., Puri & Zarutskie, 2012; Siqueira et al., 2018). While a significant stream of research on CBCs has used small samples, our sample covers about 25% of the European population of CBCs that is equivalent to Parker et al.,’s (2019) large-scale study of North American CBCs that captured about 27% of that population. An advantage to the European research setting is that small privately held firms report relatively detailed financial accounts (Vanacker et al., 2017).

Our results show that in models with main effects only there is no significant effect of leverage on sales growth and employment costs. However, when we add interactions between leverage and CBC, we find the expected negative relationship between leverage and sales growth as well as a positive relationship between leverage and employment costs for CCFs. The interaction term shows that the effect of leverage is statistically significantly weaker for CBCs relative to CCFs. A graphical analysis of the interaction effect further illustrates that there is a negative relation between leverage and sales growth for CCFs but not for CBCs. Moreover, the positive relation between leverage and employment costs for CCFs does not extend to CBCs. Overall, these empirical results show that CBCs—unlike matched CCFs—do not suffer from lower sales growth or higher employment costs as leverage increases.

Our study contributes to the CBC, business ethics, and finance literatures. First, we contribute to the burgeoning literature on CBCs. These studies have examined the motivations behind why firms become CBCs (Kim et al., 2016), their propensity to engage in diversification (Fosfuri et al., 2016), and their efforts to provide thought leadership on sustainability (Stubbs, 2017a). Additionally, studies have examined the implications of being certified as B Corp for firms’ engagement in prosocial activities (Conger et al., 2018), promoting certification (Gehman & Grimes, 2017), strengthening of CBCs’ sense of distinctiveness (Grimes et al., 2018), shifting entrepreneurial practices and orientation over time (e.g., Pollack et al., 2021; Sharma et al., 2018). Further, several studies have focused on the financial implications of being a CBC, specifically, how being a CBC affects growth and productivity (Chen & Kelly, 2015; Parker et al., 2019; Romi et al., 2018). To the best of our knowledge, we are the first to investigate the unique effects of CBCs’ financial structures versus those of the matched CCFs. Such an investigation is important because finance can be a key enabler, but also a possible constraint, for firms to realize their ambitions.

Moreover, the literature on CBCs has focused on the advantages and disadvantages of certification. For example, B Corp certification permits firms to credibly communicate their social and environmental goals to customers, employees, and other stakeholders (Cao et al., 2017; Villela et al., 2021). Thereby, CBCs can more easily attract specialized resources, such as talented employees (Conger et al., 2018). However, others show that the B Corp certification entails internal re-organization costs and decreases short-term sales growth (Parker et al., 2019). We present a novel advantage of the B Corp certification that hitherto studies have not explored. We find that relative to CCFs, the B Corp certification “shields” CBCs from the negative effects of leverage. These findings are not driven by CBCs using less leverage. Indeed, CBCs have similar leverage ratios compared to their common commercial peers. These findings are important to the financial management of CBCs.

Second, our study also generates important contributions to business ethics literature by extending knowledge on the consequences of “moral identity”. The concept of moral identity represents individuals’ commitment to behavior that fosters the well-being of others, which is also the basis for virtue and ethics at the organizational level (Weaver, 2006). Company leaders and employees make a moral identity choice when they decide to be members of CBCs, which challenges the ideology of shareholder value maximization (Kim & Schifeling, 2022). Extant research emphasizes that moral identity generates benefits, such as ethical decision making, community service, charity donations, and prosocial activities (Gu & Neesham, 2014; Neesham & Gu, 2015). A novel contribution from our study is that moral identity externalized via a B Corp Certification also brings benefits *in return* to CBCs. In this way, our study implies that business ethics has an important role in serving as a foundation for scholars to adjust finance theories to the context of CBCs. Specifically, our research suggests that scholars can contextualize theories by considering how the B Corp movement influences the moral identity and prosocial behavior of internal and external stakeholders who are committed to supporting the mission of B Corps.

Additionally, our study contributes to prior research in the business ethics domain (Santos, 2012; Yitshaki et al., 2022) by showing that CBCs tend to have advantages *despite* focusing on value creation for stakeholders instead of capturing value for the company. Past research has highlighted the idea that social enterprises tend to generate positive externalities by maximizing on value creation while only satisficing on value capture (Santos, 2012). This is a critical point because the existing literature often defines social entrepreneurs as those focusing on creating social value (Yitshaki et al., 2022) rather than on capturing value for the organization. Our study brings a novel contribution to this literature by showing evidence that CBCs, even though they generate positive externalities, tend to have financial *advantages*. Consequently, a contribution that is new from our study is that researchers can develop theoretical expectations of financial *advantages* for social enterprises like CBCs even though their focus is *not* on value capture.

Third, we contribute to the finance literature. Our study shows that the traditional theoretical perspectives from finance (e.g., Berk et al., 2010; Maksimovic & Titman, 1991)—that draw on the idea that people are self-interested actors that maximize their own personal economic gain—do not generalize to social hybrid firms, and CBCs more specifically. By contrast, our study shows that CBCs, which credibly signal their commitment to social and environmental goals (next to profit generation) to stakeholders, experience fundamentally different consequences of critical aspects of their financial structure. Overall, our study calls for more research that infuses an ethics perspective in the finance literature, and by doing so incorporates the reality that a growing group of firms have goals that transcend profit maximization but also focus on addressing social and environmental challenges “aimed at securing a good life” (and want to credibly commit to such goals).

## Theory and Hypotheses

### The Effects of Leverage: The “Stylized Facts” from Finance Theory

Debt financing has important implications for firms’ (prospective) customers and employees. If a firm takes on a loan, it has the legal obligation to make regular interest payments and pay back the principal. If the firm cannot meet these obligations, then it faces financial distress and might ultimately be forced into bankruptcy, which can lead to significant costs for customers and employees (Titman, 1984). However, when firms use equity, they are not legally obliged to pay dividends and cannot be forced into bankruptcy. So, why would entrepreneurs then use debt? First, entrepreneurs typically do not have sufficient personal funds to originate and grow their firms, and hence, access to debt financing is essential to realize their ambitions and/or their organization’s goals (e.g., Robb & Robinson, 2014; Siqueira et al., 2018). Second, for entrepreneurs, leverage brings additional personal benefits. For instance, interest payments shield firms’ profits from taxes (Vanacker & Manigart, 2010) that allows entrepreneurs to distribute more profits to themselves. Also, when entrepreneurs use debt, they need to contribute fewer personal funds. Accordingly, all else being equal, they can generate higher returns on their smaller equity contributions. Taken together, the costs and benefits of debt financing are different for entrepreneurs vis-à-vis their stakeholders, such as customers and employees.

#### Leverage and Sales Growth

If a firm goes bankrupt, customers may, for example, (expect to) lose product support, warranties, and other contractual arrangements, and/or face higher maintenance costs (Hortaçsu et al., 2013; Titman, 1984). However, customers do not only experience significant disadvantages in case of bankruptcy. Customers may also experience disadvantages from higher leverage long before bankruptcy and even in cases when firms never go bankrupt. Maksimovic and Titman (1991) show theoretically that more highly leveraged firms have an incentive to decrease product quality, possibly even at the expense of losing reputation in the future. This effect is especially strong if a leveraged firm has problems meeting its debt obligations. In line with Maksimovic and Titman (1991), Matsa (2011) shows that higher financial leverage is associated with a decline in product quality in the supermarket industry. Kini et al. (2017) find that higher leverage is associated with more frequent product recalls, which are commonly due to safety defects. Because higher leverage can be associated with significant risks and costs for customers, they might refrain from doing business with highly leveraged firms.

Moreover, less leveraged firms may aim to drive their more leveraged—and thus more vulnerable—competitors out of the market by aggressively advertising and/or temporarily underpricing their products (Chevalier, 1995). Thereby, they try to attract the customers of these firms. In line with these theoretical claims, Opler and Titman (1994) find that sales growth is 26% lower for the most leveraged firms compared to the least leveraged ones during industry downturns.

So far the empirical research (e.g., Bae et al., 2019; Campello, 2006; Opler & Titman, 1994) has focused on analyzing the effects of leverage on publicly listed firms that are typically larger and more visible than private firms. So, why would (prospective) customers be aware of the leverage of the mostly private CBCs and CCFs studied in this paper? There are at least two reasons why: first, information intermediaries (e.g., the media, credit reporting agencies) often play an important role in bringing new information, such as a firm’s financial situation, to customers (Lee & Cho, 2005). For example, the *Financial Times* reports that firms especially monitor the credit scores of small vendors.^2^ The research on the failure process of small firms shows that many customers do proactively switch to competitors as the financial outlooks of firms decrease that thereby creates a vicious cycle for these firms by driving them into bankruptcy (Ooghe & De Prijcker, 2008). Second, even when customers are not aware of a firm’s financial health and leverage, they might experience some of the direct consequences of too much leverage such as lower product/service quality. Research has shown that customers react to service failures and lower quality product offerings (e.g., Sengupta et al., 2015). Importantly, our claims above do not require that *all* customers are aware of the firm’s *exact* leverage and related financial risk. However, a subset of customers is likely to have sufficient information to make inferences about the financial health of firms; and customers can experience some of the direct effects of high leverage (e.g., lower product/service quality), that can influence their purchasing behavior.

Taken together, a finance perspective highlights that high leverage can lead to substantial disadvantages for customers (including losing product support or decreasing product quality). Customers consequently become hesitant to purchase from highly leveraged CCFs that leads to lower sales growth. Further, competitors may also try to “steal” self-interested customers from highly leveraged firms with better financial deals (or temporary underpricing) that further hampers sales growth. Therefore, we hypothesize:

##### Hypothesis 1

Leverage is negatively related to sales growth.

#### Leverage and Employment Costs

A finance perspective further argues that as financial leverage increases and needs to be serviced, employers become more likely to not honor their implicit contracts with their employees. For example, firms with higher leverage ratios are less likely to invest in health and safety programs (Moussu & Ohana, 2016).

Moreover, employees are severely affected by increased leverage because it raises the risk of financial distress and possible bankruptcy. Employees of distressed firms experience substantial earnings losses (Couch & Placzek, 2010) through (temporary) unemployment. They often need to relocate to different industries and suffer from earnings losses due to the loss of firm or industry-specific human capital. Graham et al. (2022) have estimated that employees’ total earnings losses from bankruptcy are on average about 67% of pre-bankruptcy earnings over the following seven years.

Working at a more highly leveraged firm is hence riskier for employees. In their theoretical model, Berk et al. (2010) show that risk-averse employees require compensation for bearing this risk in the form of higher wages. Two empirical studies confirm the theoretical proposition of Berk et al. (2010). Akyol and Verwijmeren (2013) find that leverage is positively related to the average employee’s wage for a sample of publicly listed US firms and a sample of established privately held Dutch firms. Similarly, Chemmanur et al. (2013) show that leverage is associated with higher labor expenses in publicly listed US firms.

One question is whether employees are aware of the leverage of a private firm (or, more broadly, its financial prospects). First, large-scale empirical evidence shows that employees often possess valuable information about the future outlook of their firm. Huang et al. (2020) analyze predictions of their firms’ business outlook by employees in Glassdoor.com and show that their outlook is informative in predicting future financial performance. They further show that employees’ outlook is especially good at predicting bad news. Second, privately held firms in Europe are required to report financial statements, and “many firms have labor representatives via a works council and/or unions who play an important role in informing employees” (Deloof et al., 2020, p. 191). Overall, employees may certainly not be aware of the exact leverage of their firms, but they are likely to be aware of the situation when leverage increases to a high level, and ultimately this is what matters for our above claims.

Taken together, a finance perspective argues that employees in higher leveraged firms require higher wages, that increases firms’ employment costs, to compensate them for the higher risk. Thus, we hypothesize:

##### Hypothesis 2

Leverage is positively related to employment costs.

### Why are CBCs Different?

In the theoretical models from finance on which hypotheses 1 and 2 are based, (risk-averse) agents maximize their utility by optimizing their personal wealth (Berk et al., 2010; Titman, 1984). In contrast, entrepreneurs in social hybrid firms, such as CBCs, have important social and environmental goals along with profit generation—and explicitly aim to create a positive impact on society (Conger et al., 2018; Doherty et al., 2014; Moroz et al., 2018). Their customers and employees usually share these goals (Austin et al., 2006; Bianchi et al., 2020; Conger et al., 2018). Consequently, entrepreneurs in social hybrid firms and their customers and employees do not primarily obtain utility from the self-serving, individualistic behavior as described in traditional finance perspectives. Social hybrid entrepreneurs are more likely to direct “their efforts toward organizational, rather than personal objectives” and “place high importance on collaboration, trust in the community and a long-term orientation” (Short et al., 2009, pp. 175–176). Tina Bhojwani, co-founder and CEO of AERA—a CBC since 2021—, indicated that “A long-term vision of giving back to the community and society as a whole provides the greater purpose and increases both employee and customer loyalty.”^3^ We expect that the utility functions and behaviors of entrepreneurs, employees, and customers at CBCs are *on average* more strongly affected by social and environmental goals than those of stakeholders in CCFs, but there might be exceptions to that. Of course, there can also be firms, which have a strong focus on their social impact, but for some reason choose not to get certified.

Consistent with the view that CBCs have an exceptionally strong social focus, Stubbs (2017b, p. 332) states that in CBCs, “profits are a *means* to achieve positive social and environmental *ends*” (Stubbs, 2017b, p. 332) but these firms also choose to be formally certified. B Corp certification is offered by B Lab that is a US-based not-for-profit organization that aims to transform “our global economy from a system that profits few to one that benefits all” and business into a “force for good” (B Lab, 2022).^4^ According to Jay Coen Gilbert, a co-founder of B Lab, certified B Corps “meet rigorous standards of social and environmental performance, legal accountability, and public transparency” (Steingard & Gilbert, 2016, p. 6). To become a CBC, firms need to (1) complete an impact assessment, (2) explicitly add their social and environmental goals to the charter, and (3) become a member and pay membership fees that depend on their sales (Romi et al., 2018). B Lab evaluates and audits the firm’s assessment, and if the firm credibly earns at least 80 out of 200 points, the certification is awarded (B Lab, 2022; Romi et al., 2018).^5^ This process can be very costly. It requires not only providing substantial documentation to and communication with B Lab but may also require operational changes like hiring new employees, changing suppliers or production processes, and consuming a large amount of managerial attention (Parker et al., 2019). Accordingly, B Corp certification is associated with a decrease in short-term sales growth (Parker et al., 2019). Thus, this evidence raises the question of why firms choose to become B Corp certified.

There are at least two reasons why: first, becoming a CBC strengthens the firm’s identification with its existing social and environmental goals and can lead to future improvements. Gehman and Grimes (2017) find that the most commonly stated reasons for becoming a CBC were that the certification was (1) aligned with the firm’s existing mission, values, purpose, or identity; (2) validated the firm’s sustainability commitment; and (3) helped firms to improve. Similarly, Conger et al. (2018) find that entrepreneurs view the certification as a “legitimating affirmation of the company’s prosocial efforts” (Conger et al., 2018, p. 8) Second, firms can use the certification as a credible signal of their social and environmental commitment to customers, employees, and other stakeholders (Parker et al., 2019). In Conger et al. (2018), for example, entrepreneurs report that B Corp certification helped them to attract talent.

Overall, CBCs are a type of social hybrid firm (e.g., Moroz et al., 2018) that like other for-profit social firms aims to do good. In addition, however, CBCs have chosen to go through a costly certification process, which enhances their identification with their non-financial goals and signals the authenticity of these goals to their customers and employees (Chauhan & O’Neill, 2020). In line with their prosocial mission, CBCs founders’ and managers’, as well as employees’ and customers’ behavior, will be consistent with the intention to have a positive impact.

### The Different Effects of Leverage in CBCs and Matched CCFs

We expect that the effects of leverage in CBCs cannot simply be described by the standard predictions of the finance perspective that we used to develop hypotheses 1 and 2. Instead, we need to develop new hypotheses that incorporate the prosocial focus of CBCs and their stakeholders.

#### Leverage and Sales Growth in CBCs Versus CCFs

Even if CBCs can also experience financial distress because of higher leverage, entrepreneurs should be less likely to abandon their firms even when that becomes financially optimal (Titman, 1984). Certainly, CBCs care about profit but it is unlikely a key motivator; rather, other elements such as “building a legacy”, “commitment to clients and community”, and “adding value to members” are key goals (Conger et al., 2018). These goals can only be achieved if the CBC continues to exist. Hence, we expect that the entrepreneurs of CBCs are oriented for the longer term to ensure their business remains operational, even if that is not financially *optimal* from their own perspective. Accordingly, customers of CBCs with higher leverage should be less concerned about the risk of losing product support or warranties.

In contrast to profit-focused CCFs (e.g., Kini et al., 2017; Maksimovic & Titman, 1991), CBCs with higher leverage are less likely to decrease the safety or quality of their products or services, even if that is a financially optimal strategy. Decreasing safety or quality to maximize profits could be at odds with their non-financial goals of creating value for society and their customers (Conger et al., 2018). Thus, CBCs are expected to act as “stewards” toward their customers, and customers of CBCs need to be less concerned that the safety or quality of products will decrease as leverage increases. However, some CBCs may decrease that quality if it allows them to help more people. But, as we detail below, customers in CBCs may be more forgiving of such behavior.

CBCs should also behave differently toward their “competitors” than predicted by standard models for CCFs that focus on maximizing financial value. The social value of CBCs activities can actually be greater if more organizations serve a certain need (Lumpkin et al., 2013). Aiming for a “sustainable solution” instead of a “sustainable advantage” for-profit social entrepreneurs should be more welcoming toward competitors (Santos, 2012, pp. 345–346). Lumpkin et al. (2013) point out that the nature of many social opportunities requires entrepreneurs to collaborate with various stakeholders, such as other for-profit social firms. Different from the behavior observed from profit-focused CCFs (Opler & Titman, 1994), CBCs are likely to not be inclined to use aggressive advertising or underpricing strategies to drive highly leveraged competitors out of the market to attract their customers. Such behavior would be at odds with CBCs’ non-financial goals and the necessary collaborative environment. Moreover, customers of CBCs, who share the same goals, can be more loyal and thus unlikely to switch suppliers for a better financial deal only.

Furthermore, not only the entrepreneur’s but also the customer’s perspective is different in for-profit social firms. Customers of for-profit social firms value and share their suppliers’ social and environmental mission (Bianchi et al., 2020; Fosfuri et al., 2016). Therefore, instead of focusing only on their own interests, they might be inclined to act as stewards and keep supporting the business with their purchases when the leverage (and risk) of the firm increases. Research on Ben & Jerry—a CBC since 2012—shows consumers are two-and-a-half times more loyal to companies that have a social purpose and incorporate values-driven action authentically throughout their business (Wharton Social Impact Initiative, 2017).^6^ Thus, given the common social goals and importance of the long-term survival of the business, we expect that there is reciprocal support between customers and suppliers.

Taken together, the classical predictions from the finance perspective are likely more pertinent to profit-focused CCFs and their customers. In these models, high leverage is increasingly expected to lead to substantial disadvantages for customers that make them hesitant to purchase from highly leveraged CCFs, which leads to lower sales growth. Further, competitors may also attract customers from highly leveraged firms with better financial deals like temporary underpricing that hampers sales growth. In contrast, taking a prosocial perspective, we expect that CBCs and their customers, who are more likely to have important, authentic social and environmental goals (Moroz et al., 2018) are less likely to engage in the same behaviors as described for their peers in CCFs. Thus, we hypothesize:

##### Hypothesis 3

The negative relation between leverage and sales growth is weaker for CBCs than for CCFs.

#### Leverage and Employment Costs in CBCs Versus CCFs

Employees in CCFs are typically focused on their financial compensation (Berk et al., 2010). However, employees in firms with social missions often “place considerable value on non-pecuniary compensation from their work” (Austin et al., 2006, p. 3). The firm’s social mission can act as a non-financial incentive for employees (e.g., Conger et al., 2018; Doherty et al., 2014). Preston (1989) develops a model in which employees at firms with social missions gain utility from their wages *and* the external social benefits generated by the firm. Past studies have measured the utility of employees by focusing on their satisfaction and well-being in social and commercial firms. Benz (2005) finds that employees are significantly more satisfied with their jobs in social rather than in CCFs. Binder (2016) even shows that working at social firms increases the well-being and life satisfaction beyond the work environment. We expect these effects to be particularly strong in CBCs as Chauhan and O’Neill (2020) find that certification further strengthens employees’ identification with the CBCs’ goals. One of the employees in the study even reported that B Corp certification was a reason she chose to work for that specific firm because “in the world of very murky definitions of social impact, B Corp is a guarantee that (…) investments are (…) not just socially responsible, but (…) impactful” (Chauhan & O’Neill, 2020, p. 23).

Overall, taking a prosocial perspective, non-pecuniary motives, that is, the aim to do good for society and the environment, are primary motivators for CBCs and their employees. Employees in CBCs have reason to expect that their employers will act as stewards and take their situation into account in financial decisions. Therefore, employees in CBCs are less likely to be risk-averse and wage-focused as predicted by financial theories and that have been observed in CCFs (Berk et al., 2010). Thus, we hypothesize:

##### Hypothesis 4

The positive relation between leverage and employment costs is weaker for CBCs than for CCFs.

## Method

### Data Sources and Sample

To test our hypotheses, we used a sample of European privately held CBCs and matched CCFs. The final dataset was a combination of data from data.world and Orbis Europe. First, we consulted the B Corp Impact Data that were made available by B Lab on data.world (https://data.world/blab/b-corp-impact-data). B Lab confirmed to us that its data comprised the population of all CBCs in Europe. We identified 548 European CBCs that were initially certified between 2013 and 2018. These firms were active in 21 European countries.^7^ We only selected European countries with more than 10 CBCs to ensure a sufficient number of firms within each country.^8^ This step eliminated only 36 firms.

Second, as the B Corp Impact Data only provided the names of CBCs, we hand collected relevant company identifiers, such as registration numbers, by looking at the respective websites of CBCs and sometimes contacting them to get more identifier information. Based on the identifiers, we used the Orbis Europe database in the next steps. This database was compiled by the Bureau van Dijk (BvD), which is a Moody’s Analytics company and is one of Europe’s leading electronic publishers of business information (Paeleman & Vanacker, 2015). The Orbis Europe database comprised financial data for publicly and privately held European firms. BvD collected information from sources that included official registers and regulatory bodies (e.g., Companies House in the UK), annual reports, private correspondence, firm websites, and news reports. BvD harmonized the financial accounts to enable accurate cross-country comparisons.

Third, to focus as clearly as possible on the different effects of leverage on CBCs versus CCFs, we used matched pairs. The sample of CBCs was matched by hand on a one-by-one basis to similar CCFs based on characteristics as of the year of B Corp certification (the CBCs in our sample are for the first time certified in the period 2013–2018). Specifically, for each CBC, we searched in the Orbis Europe database for a similar CCF in the year of certification. We used four matching criteria (e.g., Puri & Zarutskie, 2012; Siqueira et al., 2018). First, both firms needed to be active in the same country, second, in the same three-digit industry (using the NaceRev 2 industry classification), third, founded in the same year, and finally, we also required that the firms are of a similar size (i.e., total assets). We first collected the information on these characteristics for the CBCs and then we searched for a similar CCF. The matched sample approach allows us to avoid that trivial differences between CBCs and CCFs drive our findings. As King and Nielsen (2019, p. 2) detail, the matched sample approach improves our ability to draw causal inferences from real-life data, because the matching process “...amounts to a search for a data set that might have resulted from a randomized experiment but is hidden in an observational data set. When matching can reveal this “hidden experiment,” many of the problems of observational data analysis vanish”. This step resulted in omitting 194 CBCs as we did not have details on all these a priori defined characteristics for these firms. For 318 CBCs, we found a similar CCF.

Further, we required that firms had reported basic accounting data to construct our variables. In some European countries, the smallest firms were required to report an abridged balance sheet but were not necessarily required to provide a detailed profit and loss account. This requirement meant we then lacked data on sales and employment cost. Moreover, to mitigate any issues of decertification and recertification, we focused on the year of initial certification only. In the final models, we ensured that each CBC had a matched CCF. This resulted in a final sample of 136 CBCs and 136 matched CCFs from six European countries (Italy, Spain, France, the United Kingdom, Portugal, and Germany).^9^ All 272 firms were privately held firms. Our final CBC sample represented 25% of the European CBC population (initially certified between 2013 and 2018). A list with the names of the CBCs in our sample is presented in online Appendix 1.

Table 1 has a summary of the key descriptive data on the matching criteria. Table 1 shows that there are no significant differences in country distribution, industry distribution, year of certification, and age and size in the year of certification (i.e., the year of matching) between CBCs and the matched CCFs.

**Table 1:** Description of the one-to-one matched sample of CBCs and CCFs in the year of matching (i.e., year of certification)

	All firms		CBCs		CCFs		Diff. (*p*-value)
Number of firms by country							
Italy	118	43%	59	43%	59	43%	
Spain	54	20%	27	20%	27	20%	
France	50	18%	25	18%	25	18%	
United Kingdom	26	10%	13	10%	13	10%	
Portugal	20	7%	10	7%	10	7%	
Germany	4	1%	2	1%	2	1%	
Total number of firms	272	100%	136	100%	136	100%	
Number of firms by industry							
Agriculture, forestry and fishing	6	2%	3	2%	3	2%	
Manufacturing	38	14%	19	14%	19	14%	
Wholesale and retail trade; repair of motor vehicles and motorcycles	54	20%	27	20%	27	20%	
Information and communication	28	10%	14	10%	14	10%	
Financial and insurance activities	8	3%	4	3%	4	3%	
Professional, scientific and technical activities	92	34%	46	34%	46	34%	
Administrative and support service activities	12	4%	6	4%	6	4%	
Education	8	3%	4	3%	4	3%	
Human health and social work activities	6	2%	3	2%	3	2%	
Other service activities	4	1%	2	1%	2	1%	
Other	16	6%	8	6%	8	6%	
Total number of firms	272	100%	136	100%	136	100%	
Number of firms by year of certification:							
2013	2	1%	1	1%	1	1%	
2014	16	6%	8	6%	8	6%	
2015	44	16%	22	16%	22	16%	
2016	90	33%	45	33%	45	33%	
2017	68	25%	34	25%	34	25%	
2018	52	19%	26	19%	26	19%	
Total number of firms	272	100%	136	100%	136	100%	
Firm age (in years)							
Mean	12.09		12.23		11.96		0.86
S.D	12.6		12.96		12.27		
Firm size (total assets in 1000 euro)							
Mean	14,730		15,644		13,815		0.69
S.D	37,772		40,295		35,195		

Significance levels report differences between CBCs and matched CCFs using t-tests (mean)

### Variables

Below, we define the variables used in this study. Table 2 gives the descriptive statistics for all variables (except for industry and year dummies) and shows the tests on whether the means differ between CBCs and CCFs.

**Table 2:** Descriptive statistics of the full sample and two subsamples (CCFs and CBCs).

		Panel A full sample			Panel B CCFs			Panel C CBCs			*T*-tests (mean)
		N	Mean	S.D	N	Mean	S.D	N	Mean	S.D	Diff. (*p*-value)
1	Sales growth	244	1.73	2.43	122	1.57	2.19	122	1.90	2.64	0.30
2	Employment costs^a^	212	6.12	2.28	106	6.00	2.17	106	6.24	2.38	0.45
3	Leverage	272	0.16	0.21	136	0.15	0.20	136	0.18	0.22	0.17
4	CBC	272	0.50	0.50	136	0.00	0.00	136	1.00	0.00	–
5	Tangibility	272	0.14	0.20	136	0.15	0.21	136	0.12	0.18	0.26
6	Size^a^	272	7.31	2.28	136	7.30	2.26	136	7.32	2.30	0.93
7	Intagibility	272	0.08	0.16	136	0.06	0.14	136	0.10	0.17	0.03
8	Age^a^	272	2.20	0.86	136	2.19	0.86	136	2.20	0.87	0.94

Due to missing data, the number of observations for the variables sales growth and employment costs are 122 and 106, respectively, in both subsamples

^a^Log-transformed variable. This table illustrates differences between matched CCFs and CBCs using t-tests (mean). P-values are reported

#### Dependent Variables

To test Hypothesis 1, we measure *sales growth* as the total sales in year t divided by the total sales in year t-1 (e.g., Zheng et al., 2015). This measure reflects the market acceptance of a firm’s products. An increase (decrease) in sales growth shows that customers increasingly purchase (abandon) the firm’s products (Bae et al., 2019). Table 2 shows that sales growth is rather high in our full sample at 73% but with significant variation in which some firms have much lower growth rates, while others have very high growth rates. Table 2 also shows that there are no significant differences in sales growth between the subsamples of CCFs and CBCs. This finding is interesting because scholars have argued that some CBCs are not fundamentally concerned about “growth”. Furthermore, sales growth could be important because it reflects the CBCs’ ability to scale their solutions to address grand challenges, and a sales decrease is a strong indicator of customer-driven losses that indicates a decreasing impact of CBCs.

To test Hypothesis 2, we measure *employment costs* (Vanacker et al., 2013) as the natural logarithm of the total cost of employees. Wages represent the largest part of the employment costs, but our measure captures all costs for the firms, and thus also includes, for example, pension-provision costs. Table 2 shows that the average of our employment costs variable equals 6.12 in the full sample that corresponds to an average (untransformed) employment cost of 4454 (in thousand Euros). Table 2 also shows that there are no significant differences in employment costs between the subsamples of CCFs and CBCs.

#### Independent and Moderator Variables

As an independent variable, we have added *leverage* that is measured as long-term debt (i.e., debt with a maturity over one year) on total assets (e.g., Gomez-Mejia et al., 2010). Table 2 shows that some 16% of assets are financed by long-term debt. This amount is non-trivial. Interestingly, the standard deviation is also large that indicates there is significant variability in the use of long-term debt in our sample. Table 2 further shows that there are no significant differences in leverage between CCFs and CBCs.

By focusing on long-term debt, we mitigate any concerns about reverse causality from our dependent variables to leverage. For instance, a reduction in sales growth or an increase in employment costs could force firms to attract more debt to cover expenses. However, as several scholars have demonstrated, long-term debt is unlikely to be adjusted in response to short-term downfalls in performance (e.g., Bae et al., 2019). As such, our approach ensures theoretical and empirical consistency with other studies.

*CBC* is a moderator variable that is measured as a dummy variable equal to one if firms are CBCs and zero otherwise (i.e., the matched CCFs). We estimate models with leverage, CBC, and the interaction between leverage and CBCs (*leverage x CBC*). The interaction effects will show us whether CBCs experience significantly different consequences of leverage, relative to matched CCFs.

#### Control Variables

We control for other firm, industry, and year variables that may influence both our dependent and independent variables.^10^

We have also added multiple firm-level controls. Firms may invest in more tangible assets and such investments could influence their sales growth. Moreover, capital intensive firms are less likely to default that can allow them to pay lower wages (Akyol & Verwijmeren, 2013). We control for *tangibility* that is measured as tangible fixed assets (property, plant, and equipment) over total assets (Siqueira et al., 2018). The fact that size affects the growth of a firm is also well established (e.g., Vanacker & Manigart, 2010). Larger firms have, all else being equal, more employees and thus higher employment costs. We control for *size* that is measured as the natural logarithm of total assets (Siqueira et al., 2018). Next, *intangibility* is measured as the ratio of intangible assets (including R&D expenses and the value of patents, trademarks, and brands) to total assets (Paeleman et al., 2017). By virtue of their inherent inimitability, these are critical sources that may foster a competitive advantage for firms. The age of a firm is also related to its growth (Vanacker & Manigart, 2010). Therefore, we control for *age* that is measured as the natural logarithm of the number of years since formal incorporation plus one.

The industries in which firms operate may also significantly influence their employment costs and growth patterns. We, therefore have added *industry dummies* to our models to control for potential industry effects. Moreover, to control for time-related effects, we have created *year dummies* for the accounting years covered in the dataset.

### Estimation

We estimated our models using generalized linear models (GLM) for sales growth and employment costs as dependent variables. Specifically, we report GLM models that use a Gaussian (normal) distribution with an identity link function. Furthermore, we use robust standard errors clustered by country in all our regressions.

## Results

The online Appendix 2 presents the correlations between all variables used in the analyses, except for industry and year dummies. The variance inflation factors (VIFs) are well below the critical threshold of 10 and hence do not indicate problems with multicollinearity.

Table 3 presents the GLM models for sales growth (Model 1, 2, and 3) and employment costs (Models 4, 5, and 6). Model 1 and 4 present the models with only the control variables. In Models 2 and 5, we add the independent variable (i.e., leverage) and moderator variable (i.e., CBC) to these baseline models. In Model 3 and 6, we add the interaction between leverage and CBC.

**Table 3:** Results of GLM analyses for sales growth and employment costs (log-transformed)

	Sales growth			Employment costs		
	Model 1	Model 2	Model 3	Model 4	Model 5	Model 6
Intercept	4.038*** (0.283)	3.997*** (0.309)	4.134*** (0.356)	− 1.813** (0.736)	− 2.037*** (0.616)	− 1.906*** (0.563)
Tangibility	− 1.457*** (0.551)	− 1.384** (0.592)	− 1.319*** (0.418)	− 0.802** (0.332)	− 0.806** (0.372)	− 0.949* (0.487)
Size	− 0.076 (0.064)	− 0.074 (0.062)	− 0.065 (0.057)	0.846*** (0.056)	0.846*** (0.055)	0.847*** (0.050)
Intangibility	4.212* (2.389)	4.232* (2.483)	4.133* (2.315)	− 1.266*** (0.419)	− 1.450*** (0.545)	− 1.406** (0.566)
Age	− 0.631*** (0.127)	− 0.643*** (0.119)	− 0.640*** (0.110)	0.434*** (0.129)	0.428*** (0.127)	0.431*** (0.122)
Leverage		− 0.272 (1.004)	− 1.959*** (0.670)		0.238 (0.314)	1.065*** (0.186)
CBC		0.161* (0.123)	− 0.292*** (0.106)		0.228* (0.164)	0.441*** (0.106)
Leverage X CBC			2.927*** (0.858)			− 1.393*** (0.566)
Industry dummies	Yes	Yes	Yes	Yes	Yes	Yes
Year dummies	Yes	Yes	Yes	Yes	Yes	Yes
No. of observation	244	244	244	212	212	212
Log Pseudolikelihood	− 529.9	− 529.7	− 527.5	− 326.8	− 325.6	− 324.2

One-tail for IV effects. Two-tail for controls. Robust standard errors are in parentheses, clustered by country. *** *p <* 0.01, ** *p <* 0.05, * *p <* 0.10

Hypothesis 1 stated that leverage is negatively associated with sales growth. Our results in Model 2 show a negative but statistically nonsignificant direct effect of leverage. Therefore, we do not find support for Hypothesis 1. The positive and statistically significant direct effect of CBCs indicates that they have a more positive growth in sales compared to CCFs.

Hypothesis 2 stated that leverage is positively associated with employment costs. Our results in Model 5 show a positive but statistically nonsignificant direct effect of leverage. Therefore, we do not find support for Hypothesis 2. The positive and statistically significant direct effect of CBCs indicates that they have higher employment costs compared to CCFs.

**Fig. 1:**
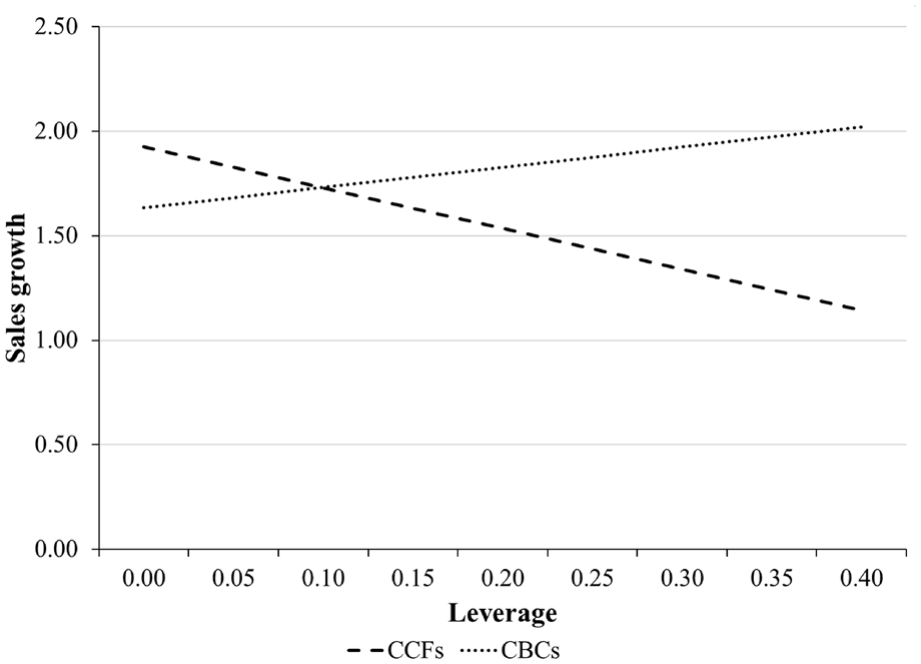
Moderating effect of CBCs on the relationship between leverage and sales growth

In Hypothesis 3, we predicted that the negative relation between leverage and sales growth was weaker for CBCs than for matched CCFs. We test this prediction by adding the interaction between leverage and CBC in Model 3. The results show a positive and statistically significant interaction effect (*β =* 2.927, *p <* 0.01). This effect shows that the negative relation between leverage and sales growth is weaker for CBCs than for CCFs. The interaction term is also plotted in Fig. 1. The figure shows that CBCs and CCFs experience different consequences for increasing leverage. The interaction effect is economically significant. Figure 1 shows that as a CBC moves from having no leverage to the mean level of leverage (0.16), sales growth slightly increases from 63 to 79%. As a CCF moves from having no leverage to the mean level of leverage, sales growth substantially decreases from 93 to 61%. Taken together, we find strong supporting evidence for Hypothesis 3.

**Fig. 2:**
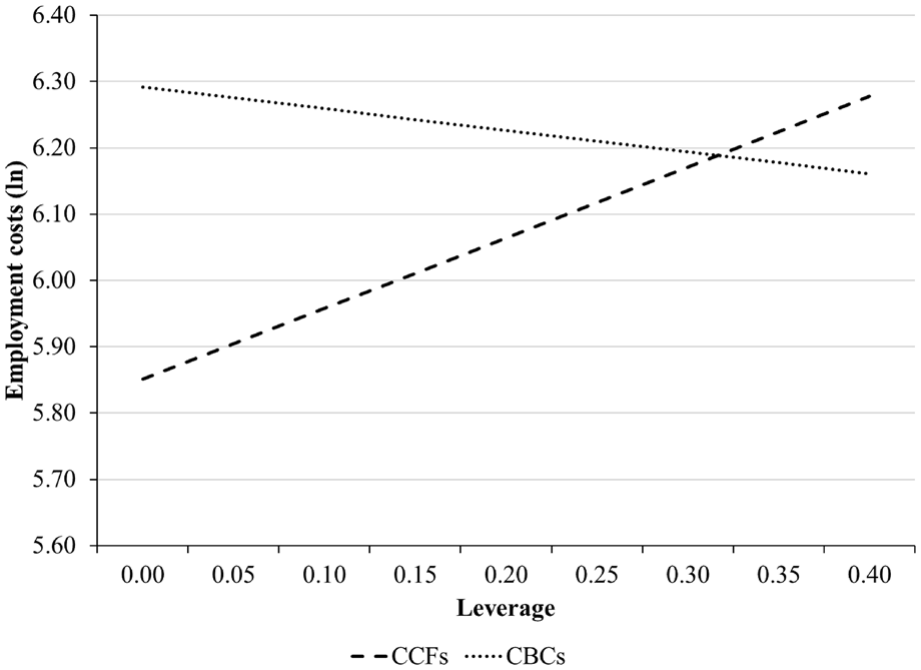
Moderating effect of CBCs on the relationship between leverage and employment costs

In Hypothesis 4, we predicted that the positive relation between leverage and employment costs was weaker for CBCs than for matched CCFs. We test this prediction by adding the interaction between leverage and CBC in Model 6. The results show a negative and statistically significant interaction effect (*β = *− 1.393, *p <* 0.01). This effect shows that the positive relation between leverage and employment costs is weaker for CBCs than for CCFs. The interaction term is also plotted in Fig. 2. Once again, this figure shows that CBCs and CCFs experience different consequences of increasing leverage. The interaction effect is also economically significant. Figure 2 shows that as a CBC moves from having no leverage to the mean level of leverage (0.16), employment costs marginally decrease from 6.29 to 6.24. In absolute monetary terms, these numbers represent a decrease in total employment costs from 540 to 513 thousand Euros at the firm level. As a CCF moves from having no leverage to the mean level of leverage, employment costs substantially increase from 5.85 to 6.02. These values represent an increase from 348 to 412 thousand Euros. Taken together, we find strong support for Hypothesis 4.

### Robustness Checks

We have conducted several additional analyses to assess the robustness of our findings.

#### Alternative Leverage Measures and Econometric Approaches

We have measured leverage as a continuous variable. However, one can argue that the probability and costs of financial distress can remain negligible when leverage remains low but that they increase dramatically only when leverage moves beyond a normal threshold (i.e., leverage is too high). Therefore, we use a first alternative measure of high leverage that is defined as a dummy variable equal to one if the firm’s leverage is above the mean and zero otherwise. A second alternative measure of high leverage is even stricter and is defined as a dummy variable equal to one if the firm’s leverage is above the mean plus one standard deviation and zero otherwise. When using these alternative measures, we find similar results. Specifically, our results are driven especially by firms with high (above mean) or very high (above mean plus one standard deviation) leverage ratios. As further robustness checks, we run OLS estimates and find similar results. As such, our results are robust to alternative econometric approaches. Furthermore, we run our models with country dummies and again find consistent results.

#### Endogeneity

Although we have used a matched sample to probe causality, we cannot prove causality (e.g., Puri & Zarutskie, 2012). Therefore, we examine the potential for endogeneity in our models by using the robustness of inference to replacement (RIR) approach (Busenbark et al., 2022). This approach makes counterfactual changes to the data and “provides insight into the percentage of a parameter estimate that would need to be biased in order to invalidate causal inference…” (Busenbark et al., 2022, p. 23). In other words, “the RIR can indicate how much of a given effect size must be biased in order to overturn an otherwise statistically significant parameter estimate” (Busenbark et al., 2022, p. 44). The resulting interpretation can account for all sources of bias from any source of endogeneity and is not limited to omitted variables only (Frank et al., 2013). For the interaction in hypothesis 3, we find that 51.60% of the estimate would have to be due to bias to make our results insignificant. This bias corresponds to 126 cases that would have to be replaced with cases for which there is a zero effect. For the interaction in Hypothesis 4, we find that 32.87% of the estimate would have to be due to bias to make our results insignificant. This percentage corresponds to 70 cases that would have to be replaced with cases for which there is a zero effect. Given the remedial measures we have already taken (i.e., matched sample approach), the RIR results indicate that the bias from endogeneity has to be very large to drive our results.

#### Verification of CCFs

CBCs formally commit to social and environmental goals beyond financial goals. We match these CBCs with similar CCFs. To verify if the matched CCFs do not hold any other sustainability certification or commitments such as, for instance, the Fair Trade Certification or Pledge1%, we manually checked their websites in March, 2022. Out of the 136 CCFs in our sample, we found the website of 120 firms (for some firms we could not find the company website due to, for example, failure). In *only* 4% of the cases, the websites reported other types of certification labels (although these were mostly product quality labels). Overall, this additional validation increases our confidence that the matched CCFs generally lack other social and/or environmental certifications.

## Discussion and Conclusion

CBCs have emerged as a rapidly growing global movement that addresses grand societal challenges (Branzei et al., 2018; George et al., 2016; Pollack et al., 2021). There is growing interest in the financing of firms that are oriented toward a social and environmental impact (e.g., Bruton et al., 2015; Markman et al., 2016) but, to date, our understanding of the effects of leverage in these firms is very limited. Still, leverage can be a double-edged sword: Debt can allow firms to form and grow to generate more of an impact, but it can also create costs for stakeholders such as customers and employees. In this study, we examine the consequences of leverage in CBCs compared to CCFs.

We find that the relations between leverage and sales growth and leverage and employment costs are significantly different across CBCs and CCFs. Neither the negative relation between leverage and sales growth nor the positive relation between leverage and employment costs for CCFs extend to CBCs.

### Academic Contributions

This study generates an important set of contributions to the CBC, business ethics, and finance literatures.

We contribute to the fast-growing literature on CBCs.^11^ Past studies have often focused on the direct effects of B Corp certification for firms’ operating performance (see, e.g., Chen & Kelly, 2015; Parker et al., 2019; Romi et al., 2018). Evidence on how B Corps are financed and how the B Corp certification uniquely impacts the consequences of firms’ financial structures remains limited. Although not directly focusing on CBCs, but instead on the legal form of the benefit corporation in the US, Cooper and Weber (2021) show that some investors prefer investing in benefit corporations relative to traditional commercial firms. Focusing on the legal form of for-profit social enterprises in Belgium, Siqueira et al. (2018) find that the capital structures of for-profit social enterprises are more stable over time than the capital structures of commercial enterprises. Our study provides a first-time glimpse into the *consequences* of debt financing for CBCs. The consequences of leverage for CCFs, such as the negative relation between leverage and sales growth and the positive relation between leverage and employment costs (e.g., Akyol & Verwijmeren, 2013; Bae et al., 2019), do not generalize to CBCs. Our matched sample ensures that these differences in the consequences of leverage across both types of firms are due to the effect of CBCs having credible prosocial goals that are also visible to stakeholders.

Having a credible prosocial behavior externalized via the global B Corp certification is a visible action by CBCs’ leaders and employees that indicates their moral identity. Moral identity represents the relative importance of being a moral person as part of a person’s self-identity (Shao et al., 2008) based on values such as fairness and care for others. In this way, moral identity refers to a commitment by individuals to pursue actions that promote the well-being of others, which serves as a foundation for organizational virtue and ethics (Weaver, 2006). Individuals’ commitment to being part of a B Corp is an explicit action of self-identification with a growing global B Corp movement, which challenges the practice of shareholder value maximization and the market share of incumbent corporations using corporate social responsibility without a B Corp Certification (Kim & Schifeling, 2022). Prior research on business ethics has emphasized that moral identity is positively associated with moral behavior, community service, charity donations, and prosocial activities (Neesham & Gu, 2015). A novel contribution from our study is that moral identity externalized via a B Corp Certification also brings benefits *back* to the organization. More specifically, these benefits consist of insulation from the adverse effects of higher leverage for CBCs, relative to matched CCFs.

Thus, our study also generates an important contribution to business ethics literature by going beyond the finding that individuals’ moral identity generates benefits such as ethical decision making and moral self-regulation (Gu & Neesham, 2014). Notably, our findings extend knowledge by suggesting that moral identity via a commitment to be a part of a CBC and participate in the global B Corp movement generates advantages to the organization by easing the negative consequences of increasing financial leverage. By doing so, our study also indicates that business ethics has an important role in extending existing finance theories developed based on traditional commercial companies in order to adjust these theories to the context of CBCs. Our results suggest that scholars seeking to develop theoretical expectations for the behavior and outcomes of CBCs need new theoretical development that considers CBCs’ identity and commitment to prosocial behavior and stakeholders’ appreciation.

Additionally, our study contributes to prior research in the business ethics domain by revealing the novel finding that social enterprises like CBCs tend to have advantages despite focusing on value creation for stakeholders rather than value capture. Past research has highlighted the idea that social enterprises tend to generate positive externalities by maximizing on value creation while only satisficing on value capture (Santos, 2012), because positive externalities refer to value created for stakeholders that is not fully captured by the organization in the form of revenues. This is a central point because past research with this orientation emphasizes that social entrepreneurs focus on business activities that generate positive externalities while merely satisficing on value capture, in contrast to common commercial entrepreneurs, who focus on maximizing value capture while just satisficing on value creation (Santos, 2012).

Similarly, recent research in the business ethics literature highlighted that social entrepreneurs focus on identifying unmet social needs with the purpose of developing an innovative solution to create social value (Yitshaki et al., 2022), which again reinforces the view that social enterprises focus on creating social value and thus positive externalities, with less emphasis on capturing value for the company. Based on the existing prior literature with this orientation, researchers might develop theoretical expectations of *disadvantages* for social enterprises like CBCs by merely satisficing on value capture. However, a critical contribution that is new to this literature is our evidence that CBCs, by buffering the consequences of leverage, tend to experience *advantages*.

Thereby, our study informs the broader debate in the literature on whether social hybrid firms require new theories from those in the finance field that were originally developed for CCFs (e.g., Dacin et al., 2010; Rawhouser et al., 2019; Wry & York, 2019). Our study does indicate that CBCs—as a specific type of social hybrid firms (Moroz et al., 2018)—do experience different consequences from their financial policies than CCFs. Accordingly, new theories would need to incorporate differences in the utility functions of firms and, more specifically, their decision makers and stakeholders.

Finally, we contribute to the finance literature. Theorizing about the effects of leverage is important in the context of social hybrid firms because financing is often assumed to be critical for firms’ ability to achieve their goals. CBCs typically face a revenue growth penalty due to attention to the assessment procedures for social and environmental performance to obtain the B Corp certification (Parker et al., 2019) that constitutes a *financial disadvantage* compared to non-certified and common commercial firms. While the literature has shown that CBCs face potential disadvantages from certification (Gehman & Grimes, 2017; Parker et al., 2019), our findings show that CBCs also get a unique *financial advantage*. This advantage is in the form of being less penalized through lower sales growth or higher employment costs, relative to their common commercial peers, when they have higher leverage. Overall, our findings call for more research that adjusts the finance theories to the context of an increasing group of firms committed to multiple goals that transcend profit maximization.

### Limitations and Avenues for Future Research

The limitations of this study represent opportunities for future research. We used several countries for which we could obtain financial data needed for our analyses, but future research should evaluate more countries and other regions to develop a comprehensive understanding of CBCs worldwide. Next, we used a quantitative matched sample approach to assess the significance of differences between CBCs and CCFs, and new studies should use different methods to further explore how multiple stakeholders can support CBCs and other categories of social firms (Bacq & Lumpkin, 2014). Using other approaches, such as surveys or interviews, could be particularly valuable to corroborate our results and provide more direct evidence on the underlying mechanisms. Our study does not provide direct evidence for the mechanisms through which leverage influences sales growth and employment costs. However, similar to other business and management studies (e.g., Engelen et al., 2015), these mechanisms are difficult to measure and would require us to supplement our database with, for example, survey evidence. Future studies might collect finer-grained data and could conduct interviews with entrepreneurs, employees, and customers of CCFs and CBCs to hear about how they perceive different levels of leverage. Moreover, we have focused on traditional financial leverage ratios, but future studies should also investigate the specific sources from which CBCs raise debt financing, including banks, crowdfunding, microfinance, and peer-to-peer approaches (Bruton et al., 2015) to facilitate the attainment of their goals. Finally, the COVID-19 pandemic also provides an interesting opportunity for future research. A question would be to what extent CBCs are better insulated from the negative consequences of the COVID-19 pandemic compared to CCFs.

### Contributions for Practice

Following the growing number of CBCs, new financial instruments to support these socially responsible for-profit firms have become important at the firm, cross-sector, and policy levels in different countries (Bruton et al., 2015). For instance, at the firm level, the Force for Good Fund associated with the online crowdfunding platform WeFunder invests in “Best for the World” CBCs that score in the top 10% of CBCs worldwide (Force for Good Fund, 2019).^12^ At the cross-sector partnership level, one example is Trivident that is a Flemish Equity Fund for social firms founded in 2001 by social economy actors, the government, and the private sector. At the policy level, the European Union Programme for Employment and Social Innovation has been promoting firms whose design is sustainable to address society’s challenges and financial solutions (Book of Goodbiz, 2018).^13^

However, there is limited research to inform the development of financing solutions tailored to socially responsible for-profit firms like CBCs. This study provides new knowledge to help practitioners develop new financial solutions for for-profit social firms. Our study shows that CBCs have more favorable consequences from leverage than their common commercial counterparts. Based on our findings, bankers and other financial capital providers involved in the rise of social investing (Yan et al., 2019) should become more aware of the opportunities to serve the segment of for-profit social firms, such as by designing loans and investment options for CBCs, given that they tend to be protected from the adverse effects of higher leverage.

### Overall Conclusion

This study examines the consequences of financial leverage in CCFs versus CBCs. We show that CBCs experience less adverse effects from higher leverage, such as lower sales growth and increasing employment costs, than CCFs. Accordingly, the CBCs category is better protected from the expected negative effects of higher leverage than CCFs. In this way, our findings further show that stakeholders support the B Corp certification.

## Supplementary Information

Below is the link to the electronic supplementary material.

Supplementary file1 (DOCX 27 KB)

## Data Availability

Data relevant to this study is available upon reasonable request.

## Footnotes

1 The Benefit Corporation legal status in the United States is distinct from the B Corp Certification, which operates in multiple countries. For a detailed discussion, see Lucas et al. (2022), Rawhouser et al. (2015) or Siqueira et al. (2018).

2 https://www.ft.com/content/a9791cc6-4829-11e1-b1b4-00144feabdc0 opened on September 16, 2022.

3 https://www.iwaymagazine.com/blogs/fashion/aera-earns-b-corporation-certification opened on September 16, 2022.

4 https://www.bcorporation.net/en-us/movement/about-b-lab/ opened on September 16, 2022.

5 https://www.bcorporation.net/en-us/certification opened on September 16, 2022.

6 https://socialimpact.wharton.upenn.edu/news/podcast-ben-jerrys-rob-michalak-talks-social-mission-dollars-change/ opened on September 16, 2022.

7 These 21 countries are Austria, Belgium, Czech Republic, Denmark, Finland, France, Germany, Greece, Hungary, Ireland, Italy, Luxembourg, Malta, Netherlands, Norway, Poland, Portugal, Spain, Sweden, Switzerland, and the United Kingdom.

8 Countries with the minimum of 10 B Corps are France, Germany, Italy, Netherlands, Portugal, Spain, Switzerland, and the United Kingdom.

9 Other European countries with more than 10 B Corps were the Netherlands and Switzerland. We did try to collect data of the B Corps in these countries. However, none of them reported the basic data that was needed for this study.

10 The remaining debt capacity of firms and related costs of financial distress may also be determined by Earnings Before Interest, Taxes, Depreciations and Amortizations (EBITDA) to total assets ratios (i.e., all else being equal, firms with higher EBITDA to total assets have more cash flows available to repay fixed debt-related payments). When we control for EBITDA scaled by total assets in our basic analyses, our results remain stable, although there is a significant drop in observations due to missing information on EBITDA.

11 We do not aim here for a complete comparison to existing research on CBCs but refer the interested reader to Cao and Gehman (2021).

12 https://wefunder.com/forceforgood/ opened on September 16, 2022.

13 https://issuu.com/boljseresitve/docs/goodbiz_knjiga_web_spread opened on September 16, 2022.
