# Supplementary material for: The Consequences of Financial Leverage: Certified B Corporations’ Advantages Compared to Common Commercial Firms
Source: J Bus Ethics. 2023 Feb 14:1–17. Online ahead of print. doi: 10.1007/s10551-023-05349-5 (PMC9925939; doi:10.1007/s10551-023-05349-5)
Supplement: Supplementary file 1 — Supplementary file1 (DOCX 27 KB) [file 10551_2023_5349_MOESM1_ESM.docx]

**ONLINE APPENDIX 1** List of CBCs.

|  | **CBC name** | **Country** | **Certification year** |
| --- | --- | --- | --- |
| 1 | NATIVA S.R.L. SOCIETA' BENEFIT IN FORMA ABBREVIATA NATIVA SRL SB | Italy | 2013 |
| 2 | LG CONSEIL | France | 2014 |
| 3 | EQUILIBRIUM S.R.L. | Italy | 2014 |
| 4 | ROOTS FOR SUSTAINABILITY SL | Spain | 2014 |
| 5 | TREEDOM S.R.L. SOCIETA' BENEFIT | Italy | 2014 |
| 6 | D-ORBIT SOCIETA' PER AZIONI | Italy | 2014 |
| 7 | FRATELLI CARLI SOCIETA' PER AZIONI SOCIETA' BENEFIT SIGLABILE IN FRATELLI CARLI S.P.A. S.B. | Italy | 2014 |
| 8 | BIORUMO - CONSULTORIA EM AMBIENTE E SUSTENTABILIDADE, LDA | Portugal | 2014 |
| 9 | DISTRETTO TECNOLOGICO TRENTINO SOCIETA' CONSORTILE A RESPONSABILITA' LIMITATA | Italy | 2014 |
| 10 | AIR - AGENCE INNOVATION RESPONSABLE | France | 2015 |
| 11 | MACPHIE LIMITED | United Kingdom | 2015 |
| 12 | MA BONNE ETOILE | France | 2015 |
| 13 | PIXELIS | France | 2015 |
| 14 | EKODEV | France | 2015 |
| 15 | GENERATION INVESTMENT MANAGEMENT LLP | United Kingdom | 2015 |
| 16 | INGEUS UK LIMITED | United Kingdom | 2015 |
| 17 | LILY'S KITCHEN LIMITED | United Kingdom | 2015 |
| 18 | LOGFRAME - CONSULTORIA E FORMACAO, LDA | Portugal | 2015 |
| 19 | NATURE ET DECOUVERTES SA | France | 2015 |
| 20 | DERMOPHISIOLOGIQUE S.R.L. SOCIETA' BENEFIT O IN BREVE DERMOPHISIOLOGIQUE S.R.L. SB | Italy | 2015 |
| 21 | CUENTO DE LUZ SL | Spain | 2015 |
| 22 | EM PROJECTES DIDACTICS SL | Spain | 2015 |
| 23 | BATES WELLS & BRAITHWAITE LONDON LLP | United Kingdom | 2015 |
| 24 | RECYCLIVRE | France | 2015 |
| 25 | LIMITES A PROVA, UNIPESSOAL, LDA | Portugal | 2015 |
| 26 | WORLDCOO SL. | Spain | 2015 |
| 27 | IWOPI-HEALTH & SPORT WITH RESPONSIBILITY SL. | Spain | 2015 |
| 28 | ESTUDIO CASA CARLOTA SL. | Spain | 2015 |
| 29 | MONDORA S.R.L. SOCIETA' BENEFIT | Italy | 2015 |
| 30 | DES ENJEUX ET DES HOMMES | France | 2015 |
| 31 | MOBISOL GMBH | Germany | 2015 |
| 32 | ZORDAN S.R.L. SOCIETA' BENEFIT IN FORMA ABBREVIATA ZORDAN S.R.L. SB | Italy | 2016 |
| 33 | AGUIA CONSULTING | France | 2016 |
| 34 | ECOVERITAS SA | Spain | 2016 |
| 35 | WHEB ASSET MANAGEMENT LLP | United Kingdom | 2016 |
| 36 | STEELTER TALENT SOLUTIONS S.L. | Spain | 2016 |
| 37 | MAILWORK ECOSOSTENIBILI S.R.L. SOCIETA' BENEFIT | Italy | 2016 |
| 38 | PARADISI - S.R.L. | Italy | 2016 |
| 39 | N.& B. S.R.L. SOCIETA' BENEFIT | Italy | 2016 |
| 40 | KOIKI HOME SL. | Spain | 2016 |
| 41 | EMMERRE S.R.L. SOCIETA' BENEFIT IN FORMA ABBREVIATA EMMERRE S.R.L. SB | Italy | 2016 |
| 42 | AUTHENTICITYS EXPERIENCES SL. | Spain | 2016 |
| 43 | RIGHT HUB S.R.L. | Italy | 2016 |
| 44 | INSIEME SOCIETA' COOPERATIVA BENEFIT | Italy | 2016 |
| 45 | PATTE BLANCHE | France | 2016 |
| 46 | MARIOWAY S.R.L. SOCIETA' BENEFIT IN LIQUIDAZIONE IN FORMA ABBREVIATA MARIOWAY S.R.L. SB | Italy | 2016 |
| 47 | PASTICCERIA FILIPPI SRL SOCIETA' BENEFIT | Italy | 2016 |
| 48 | GOOD POINT S.R.L SOCIETA' BENEFIT | Italy | 2016 |
| 49 | MANAGING A SUSTAINABLE BUSINESS SL. | Spain | 2016 |
| 50 | TETERUM SL. | Spain | 2016 |
| 51 | PEOPLE WHO GLOBAL SL. | Spain | 2016 |
| 52 | DIVINE CHOCOLATE LIMITED | United Kingdom | 2016 |
| 53 | VALLI DEL BITTO S.P.A. SOCIETA' BENEFIT | Italy | 2016 |
| 54 | CASA VINICOLA LA TORRE COOPERATIVA AGRICOLA ZANOLARI SOCIETA' BENEFIT - PER BREVITA' C.V.L.T. COOPERATIVA AGRICOLA ZANOLARI SB | Italy | 2016 |
| 55 | GOGREEN STORE S.R.L. | Italy | 2016 |
| 56 | GREENAPES SOCIETA A RESPONSABILITA LIMITATA SOCIETA' BENEFIT | Italy | 2016 |
| 57 | SALCHETO S.R.L. SOCIETA' AGRICOLA SOCIETA' BENEFIT | Italy | 2016 |
| 58 | BOA ENERGIA, LDA | Portugal | 2016 |
| 59 | FOCUS LAB SOCIETA' A RESPONSABILITA' LIMITATA - BENEFIT | Italy | 2016 |
| 60 | ''PALAZZO CARPEGNA SOCIETA' A RESPONSABILITA' LIMITATA'' | Italy | 2016 |
| 61 | DAVINES S.P.A. | Italy | 2016 |
| 62 | COMETECH S.R.L. | Italy | 2016 |
| 63 | MANRESANA DE MICOBACTERIOLOGIA SL. | Spain | 2016 |
| 64 | FACILE AIUTO SOCIETA' BENEFIT A RESPONSABILITA' LIMITATA | Italy | 2016 |
| 65 | SEETEC BUSINESS TECHNOLOGY CENTRE LIMITED | United Kingdom | 2016 |
| 66 | ACTIMPACT S.L. | Spain | 2016 |
| 67 | STONE SOUP CONSULTING, LDA | Portugal | 2016 |
| 68 | ELIDRIA S.R.L. SOCIETA' BENEFIT | Italy | 2016 |
| 69 | MORE THAN HONEY, UNIPESSOAL, LDA | Portugal | 2016 |
| 70 | SIDIESE | France | 2016 |
| 71 | EXECUTIVE SERVICE S.R.L. SOCIETA' BENEFIT | Italy | 2016 |
| 72 | IMPACT HUB SRL | Italy | 2016 |
| 73 | CONCEITO F.A. - FORMACAO E ARQUITECTURA, LDA | Portugal | 2016 |
| 74 | JOJO MAMAN BEBE LTD | United Kingdom | 2016 |
| 75 | PERLAGE S.R.L. | Italy | 2016 |
| 76 | THE PURE PROJECT | France | 2016 |
| 77 | NWG S.P.A. - SOCIETA' BENEFIT IN FORMA ABBREVIATA NWG S.P.A. SB | Italy | 2017 |
| 78 | I&P | France | 2017 |
| 79 | WEKIWI S.R.L. | Italy | 2017 |
| 80 | EVOLVERE S.P.A. SOCIETA' BENEFIT | Italy | 2017 |
| 81 | GOLDMANN & PARTNERS S.R.L. SOCIETA' BENEFIT | Italy | 2017 |
| 82 | DIASEN S.R.L. | Italy | 2017 |
| 83 | WASTE IS MORE | France | 2017 |
| 84 | MEDIA RESPONSABLE SL | Spain | 2017 |
| 85 | FUSIO D'ARTS TECHNOLOGY SOCIEDAD LIMITADA. | Spain | 2017 |
| 86 | SANTA FRANCESCA CABRINI SRL SOCIETA' BENEFIT IN BREVE SFC SRL SOC IETA' BENEFIT | Italy | 2017 |
| 87 | XBRIDGE LIMITED | United Kingdom | 2017 |
| 88 | LES PRES RIENT BIO | France | 2017 |
| 89 | INVENTO INNOVATION LAB IMPRESA SOCIALE S.R.L. | Italy | 2017 |
| 90 | PALM S.P.A. SB | Italy | 2017 |
| 91 | LOCAL TO YOU S.R.L. | Italy | 2017 |
| 92 | ALTMAN PARTNERS | France | 2017 |
| 93 | INBONIS SA | Spain | 2017 |
| 94 | WAMI S.R.L. S.B. | Italy | 2017 |
| 95 | WENOW | France | 2017 |
| 96 | SORRISO E SALUTE S.R.L. | Italy | 2017 |
| 97 | FONCIERE CHENELET | France | 2017 |
| 98 | OMAL S.P.A. SOCIETA' BENEFIT | Italy | 2017 |
| 99 | ALESSI S.P.A. - SOCIETA' BENEFIT | Italy | 2017 |
| 100 | TEK S.R.L. SB | Italy | 2017 |
| 101 | HOVIONE FARMACIENCIA, S.A. | Portugal | 2017 |
| 102 | ECOTONE | France | 2017 |
| 103 | METALLI LINDBERG S.R.L. | Italy | 2017 |
| 104 | ARP ASTRANCE | France | 2017 |
| 105 | ARTATTACK GROUP S.R.L. | Italy | 2017 |
| 106 | FIRSTFLOOR S.R.L. SOCIETA' BENEFIT IN FORMA ABBREVIATA FIRSTFLOOR SRL SB | Italy | 2017 |
| 107 | INTERNATIONAL NAPOLI NETWORK - SOCIETA' COOPERATIVA DI PRODUZIONE | Italy | 2017 |
| 108 | IDEEGREEN SRL SOCIETA' BENEFIT | Italy | 2017 |
| 109 | EQUANUM | France | 2017 |
| 110 | BOTTEGA FILOSOFICA S.R.L. SOCIETA' BENEFIT | Italy | 2017 |
| 111 | GLASSY FILMS SL | Spain | 2018 |
| 112 | SPECIALISTERNE SL. | Spain | 2018 |
| 113 | IMPACT HUB SL. | Spain | 2018 |
| 114 | CODE FOR ALL, LDA | Portugal | 2018 |
| 115 | GREEN MEDIA LAB S.R.L. SOCIETA' BENEFIT OVVERO, IN FORMA ABBREVIATA, GREEN MEDIA LAB S.R.L. SB | Italy | 2018 |
| 116 | ECOALF RECYCLED FABRICS SL | Spain | 2018 |
| 117 | ABEL & COLE LIMITED | United Kingdom | 2018 |
| 118 | ALLOS HOF-MANUFAKTUR GMBH | Germany | 2018 |
| 119 | SALES S.R.L. SOCIETA' BENEFIT | Italy | 2018 |
| 120 | INTEXO SOCIETA' BENEFIT S.R.L. | Italy | 2018 |
| 121 | THE MANIPEDI, SOCIEDADE UNIPESSOAL, LDA | Portugal | 2018 |
| 122 | SPAZIO NOPROFIT S.R.L. SOCIETA' BENEFIT | Italy | 2018 |
| 123 | BRAIBOOK SL. | Spain | 2018 |
| 124 | EXPANSCIENCE | France | 2018 |
| 125 | ARS S.R.L. | Italy | 2018 |
| 126 | ABAFOODS S.R.L. | Italy | 2018 |
| 127 | CEF PUBLISHING S.P.A. | Italy | 2018 |
| 128 | JUST ASK ESTATE SERVICES LIMITED | United Kingdom | 2018 |
| 129 | COMUNIDADES NATURALES DE CONSUMO SL. | Spain | 2018 |
| 130 | INNOCENT LIMITED | United Kingdom | 2018 |
| 131 | FLOR DE DONANA SL. | Spain | 2018 |
| 132 | VEJA FAIR TRADE S A R L | France | 2018 |
| 133 | WAIKI ORGANIC FOODS SL. | Spain | 2018 |
| 134 | ADEAS RRHH SL | Spain | 2018 |
| 135 | COOPERATIVE MU | France | 2018 |
| 136 | CONNEXING | France | 2018 |

**ONLINE APPENDIX 2** Correlations.

*Notes:* Due to missing data, the number of observations for the variables sales growth and employment costs are 122 and 106, respectively. ^a^ Log-transformed variable. ^b^ Binary variable, correlations should be interpreted with care.
